# Seed Transmission of *Pseudoperonospora cubensis*

**DOI:** 10.1371/journal.pone.0109766

**Published:** 2014-10-17

**Authors:** Yigal Cohen, Avia E. Rubin, Mariana Galperin, Sebastian Ploch, Fabian Runge, Marco Thines

**Affiliations:** 1 Faculty of Life Sciences, Bar-Ilan University, Ramat Gan, Israel; 2 Institute of Botany (210), University of Hohenheim, Stuttgart, Germany; 3 Biodiversity and Climate Research Centre, Frankfurt, Germany; Agriculture and Agri-Food Canada, Canada

## Abstract

*Pseudoperonospora cubensis*, an obligate biotrophic oomycete causing devastating foliar disease in species of the *Cucurbitaceae* family, was never reported in seeds or transmitted by seeds. We now show that *P. cubensis* occurs in fruits and seeds of downy mildew-infected plants but not in fruits or seeds of healthy plants. About 6.7% of the fruits collected during 2012–2014 have developed downy mildew when homogenized and inoculated onto detached leaves and 0.9% of the seeds collected developed downy mildew when grown to the seedling stage. This is the first report showing that *P. cubensis* has become seed-transmitted in cucurbits. Species-specific PCR assays showed that *P. cubensis* occurs in ovaries, fruit seed cavity and seed embryos of cucurbits. We propose that international trade of fruits or seeds of cucurbits might be associated with the recent global change in the population structure of *P. cubensis*.

## Introduction

Downy mildew is a major disease of cucurbits [1]–[3]. The pathogen, *Pseudoperonospora cubensis* (Berk. & Curt.) Rost. (*Oomycota*, *Peronosporaceae*), attacks over 40 cucurbitaceous host species representing about 20 genera [1]. Infection occurs on cotyledons and true leaves. There are only two reports on the occurrence of this downy mildew on other plant parts. Van Haltern in the USA [4] found sporangiophores on the stem, petioles, tendrils, and peduncles of blossoms of heavily infected cantaloupe vines, but not on small fruits, and D'Ercole in Italy [5] recorded sporulation of the mildew on cucumber fruits grown under cover. There is no evidence that the pathogen spreads systemically in its hosts, nor that it is seed-borne or seed-transmitted. Here we report for the first time that *P. cubensis* may be fruit-borne, seed-borne, and seed-transmitted in cucurbits. Our preliminary report was published earlier [6].

## Materials and Methods

### Plants

Nine cucurbit species were grown in 4 net houses # 2, 3, 5 and 6 (6×50 m each) located on the north-east part of the campus, at Bar-Ilan University Farm, Israel in six seasons during 2012–2014. These species were also grown in a controlled glasshouse #3 located in the south-west part of the campus. The species were cucumber (*Cucumis sativum*, cvs.SMR-18 and Nadiojny), melon (*Cucumis melo* var. *reticulatus*, cvs. Ananas Yokneam and Ein-Dor), pumpkin (*Cucurbita maxima* cvs. Tripoli and Armonim), squash (*Cucurbita pepo* cvs.Beruti and Arlika), butternut gourd (*Cucurbita moschata* cv.Waltham), watermelon (*Citrullus lanatus* cv. Mallali), bottle gourd (*Lagenaria vulgaris*, local cultivar), sponge gourd (*Luffa cylindrica*, local cultivar) and bitter gourd (*Momordica balsamina*, local cultivar). Planting took place on February (Spring season), August (Autumn season) and November (Winter season). Plants were fertilized weekly with 0.5% N:P:K and sprayed with fungicides against powdery mildew when required. Natural infection with downy mildew occurred in net-houses # 2, 3, 5 and 6 at all seasons on leaves of all cucurbits except watermelon, sponge gourd and bitter gourd. No downy mildew showed up in glasshouse #3 due to the lack of free moisture on the leaves. Seeds for all experiments describe herein were collected from greenhouse #3. Frequent PCR assays done with seeds samples taken from glasshouse #3 were proved negative for *P. cubensis*.

The procedures describe below were all similarly applied to healthy plant material derived from greenhouse #3 and infected (symptomless flowers, ovaries, fruits, seeds) plant material derived from net-houses #2, 3, 5 and 6.

### Recovery of *P. cubensis* from flowers, fruits, and seeds

Strict hygiene measures were undertaken while attempting to recover *P. cubensis* from flowers, ovaries, fruits and seeds. Female flowers for ovaries were taken from cucumber and squash. Fruits were collected from cucumber, butternut gourd, pumpkin, bottle gourd and squash. Some fruits carried empty seeds because of lack of adequate pollination. The ovaries and fruits were washed with excessive soap water, washed thoroughly with tap water; surface sterilized by dipping in 4% hypochlorite solution for 10 minutes; dipped momentarily in ethanol and washed with sterile water. All further processing steps, including PCR, were carried out under strict sterile conditions. Ovaries, fruit seed cavity, empty seeds and mature seeds were homogenized in sterile cold water and used for inoculation of healthy detached leaves (taken from greenhouse #3). Mature seeds were sown (see below) in sterile soil mixture or used for microscopy. Three 50 µl droplets were taken from each homogenate, placed on a glass slide, covered with a cover slip, and examined with a dissecting microscope for the presence of mycelia or sporangia of *P. cubensis*. Fifty droplets, 50 µl each, of each homogenate were pippeted onto the lower surface of a detached cucumber leaf laying on a wet sterile filter paper in a 14 cm diameter Petri dish, and fifty such droplets were similarly inoculated onto a detached leaf of butternut gourd. Inoculated leaves were incubated at 20°C under 12 h photoperiod for 3 days, then washed with sterile water to remove the homogenate droplets, and thereafter kept for three weeks at 20°C under 12 h photoperiod to allow for downy mildew development. Detached leaves inoculated with droplets of sterile water served as controls. Symptoms of downy mildew with sporulation of *P. cubensis* appeared on some detached leaves inoculated with plant material (fruit seed-cavity homogenates, fruit tissue slices, ovary homogenates, and leaf pieces taken from seedlings developed from seeds) derived from net-houses #2, 3, 5 and 6 but never in similar plant material derived from greenhouse #3. Sporangia were propagated on detached leaves of cucumber laying on wet filter paper in 14 cm Petri dishes. The pathotype of the isolates was determined by inoculation of detached leaves of 9 cucurbits species as described before [7], [8]. Mating type was determined by inoculation of melon and cucumber leaf discs with sporangial mixtures of the test isolate and a tester isolate of known mating type as described before [9].

### Recovery of *P.cubensis* from hypocotyls

Seeds were surface sterilized and sown in sterile pots (60 ml) filled with a sterile soil mixture (peat: vermiculite, 1∶1, v/v), 1 seed per pot. Plants were grown in greenhouse #3 and when reached the first true leaf stage (2 weeks after sowing) their hypocotyl was removed with a sterile scalpel, surface sterilized, placed in sterile water (1 hypocotyl/5 ml) and homogenized with a sterile blade (2 minute, 7000 rpm, 4°C). The homogenate was used for inoculation of detached leaves of cucumber and butternut as described above.

### Microscopy

Free hand sections were taken with a sterile razor blade from surface sterilized ovaries or fruits. Slices were placed on detached leaves of cucumber or butternut gourd to allow infection. Other slices were boiled in ethanol for 10 minutes, placed for 24 h in basic aniline blue solution (0.05%, pH 8.9) at 4°C, stained with 0.01% calcofluor (Sigma), and examined with Olympus A70 epifluorescent microscope for the presence of sporangia and mycelia [10]. A similar procedure was employed to embryos taken from mature seeds.

### Recovery of *P.cubensis* from Seedlings

Seeds were surface sterilized, placed on sterile filter paper in sterile 14 cm petri dishes or 20×20×3 cm sterile plastic dishes (Nunk, Denmark) and incubated at 25°C under 12 h photoperiod. When cotyledons were produced (about 7 days), plants were transplanted into 0.5 L pots filled with sterile potting soil, while others were used for DNA extraction. Plants were maintained at 20°C at 12 h photoperiod to allow for downy mildew development. When symptoms appeared, plants were sealed in 1 L sterile plastic boxes (100% RH) for several days to enable sporulation of *P. cubensis*.

### DNA extraction from plants or sporangia of *P. cubensis*

The method of Tinker et al [11] was employed with modifications. Samples of approximately 100–500 mg leaf, hypocotyl, root, ovary, or fruit tissue, or a sample of about 1×10^5^ sporangia, were macerated in 1.5-ml micro-tubes using disposable pellet pestle grinders. Maceration was continued after adding 0.6 ml CTAB (hexadecyltrimethyl-ammonium bromide) buffer [1.4 M NaC1, 20 mM EDTA, 100 mM TRIS-Cl, 2% (W/V) CTAB pH 8.0], and the samples were incubated at 60°C for 45 min. The samples were then extracted with 0.6 ml chloroform/isoamyl alcohol (24∶1) and centrifuged at 12000 g for 5 min. The aqueous phase was transferred to a 1.5-ml tube where the DNA was precipitated with an equal volume of cold (-20°C) isopropanol. DNA concentration was determined with a ND-1000 spectrophotometer (NanoDrop USA). DNA separation was done on a 1.2% agarose gel and staining with ethidium bromide.

### DNA extraction from seeds

Dry seeds were placed in 2 ml tubes and rehydrated with sterile water for 15 minutes. After the water was removed, sodium hypochlorite solution (4%) containing 0.1% Tween 20 (to break surface tension) was added to the samples for 10 minutes. Seeds were rinsed with sterile water for 5 minutes. The embryo and the integument were separated and transferred to 96 well plates with 1.3 ml tubes. Care was taken not to cross-contaminate the samples and new gloves and sterile forceps were used for every seed. DNA extraction was conducted by using the BioSprint 96 DNA Plant Kit (Qiagen, Hilden, Germany) in combination with a KingFisher Flex (ThermoFisher Scientific, Waltham, USA) DNA extraction robot. The quality of the extraction was tested by conducting a PCR with the primers ITS1 and ITS4 developed by White et al. [12]. For all samples amplifiable DNA was obtained.

### Primer development and molecular detection

Species-specific primers were developed based on *cox*2 sequences of *Pseudoperonospora humuli*, *Pseudoperonospora cubensis* and related species which were obtained from the database of the National Center for Biotechnology (http://www.ncbi.nlm.nih.gov/). The primers for *P. humuli* and the two clades of *P. cubensis*, which had been reported in Runge et al. [13], as well as their specificity, are shown in Table S1. As described by Ploch et al. [14] two PCRs were carried out to detect the pathogens with high specificity and sensitivity. In the first PCR, 0.4 mM of Oomycete specific primers *cox*2-F and *cox*2-R [15] were used in a reaction mixture containing 1× Mango PCR Buffer, 0.2 mM dNTPs, 1 mM MgCl_2_, 0.8 mg/ml BSA and 0.5 U Mango Taq DNA Polymerase (Bioline, Luckenwalde, Germany). For all three primer combinations (Table S1) a separate nested PCR was conducted with a 1 to 10 dilution of the oomycete specific PCR. Cycling temperature and times are detailed in Table S2.

## Results

### Downy mildew in butternut gourd fruits

**Figure 1:**
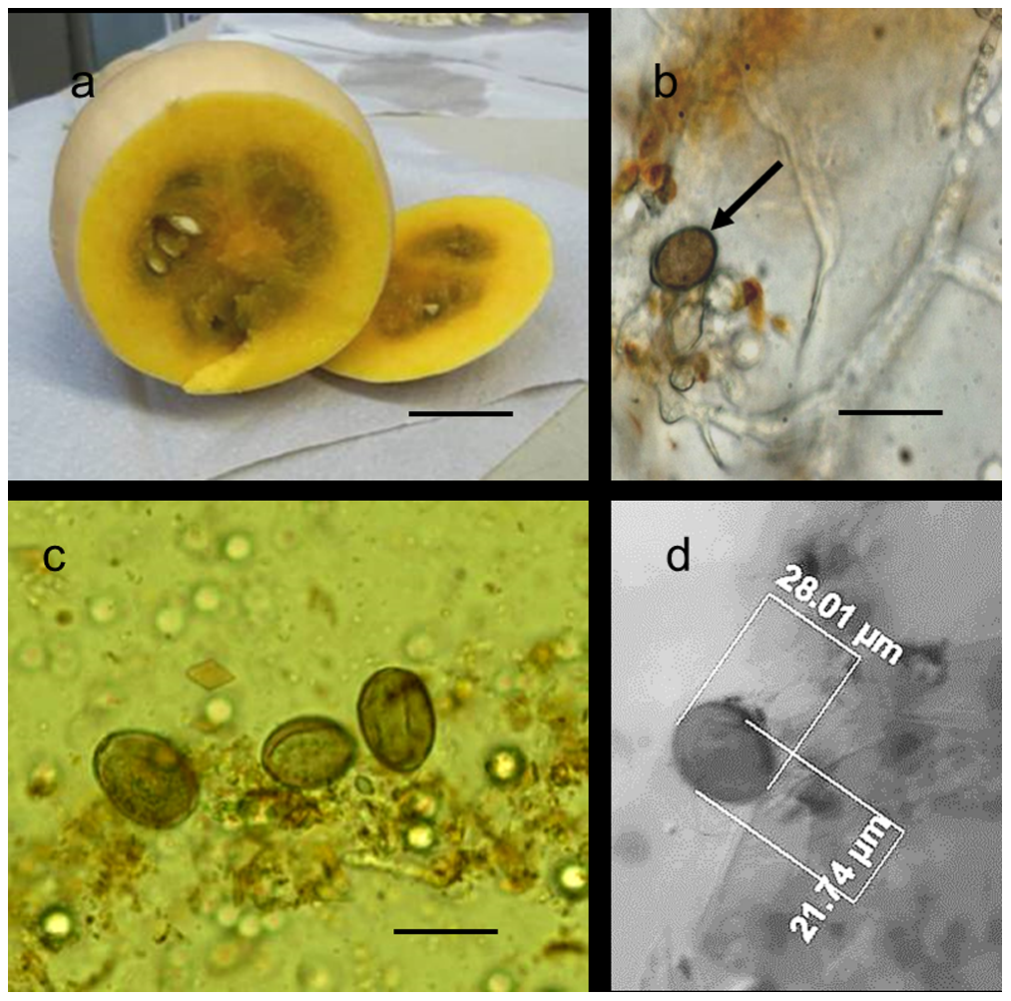
Infection of butternut gourd (*Cucurbita moschata*) fruit with *Pseudoperonospora cubensis*. **a**, transversally-cut fruit showing a dark seed cavity. **b**, **c**, **d** coenocytic mycelium with sporangia in the seed cavity. Bar in **a = **3 cm, **b = **30 µm, **c** = 20 µm.

Butternut gourd fruits taken from downy mildew-infected plants revealed a dark seed cavity (Fig. 1a). Microscopic examinations of free-hand sections taken from the seed cavity revealed coenocytic hyphae and a few sporangia of *P. cubensis* (Fig. 1 b–d). No sporangiophores of *P. cubensis* were seen. Between 0–10 sporangia per 50 µl droplet were detected in the seed cavity homogenate of various fruits. Butternut gourd fruits taken from healthy plants showed no discoloration of the seed cavity nor mycelia or sporangia.

### Recovery of *P. cubensis* from ovaries, fruits, hypocotyls and seeds

Table 1 summarizes the data obtained during 2012–14. A total of 129 ovaries of cucumber, melon or squash were collected from downy mildew-infected plants, surface sterilized, crushed and inoculated onto detached healthy leaves of cucumber and butternut gourd. No ovaries of butternut gourd were used. About 25% of the ovaries were infectious, capable of producing typical downy mildew symptoms on the detached leaves (Table 1A). The isolates recovered from ovaries of cucumber or melon belonged to pathotype 3 mating type A1, while those recovered from squash belonged to pathotype 6 mating type A2.

**Table 1:** *Pseudoperonospora cubensis* was recovered from the reproductive organs of downy mildew- infected plants (A–D) but not from the reproductive organs of healthy plants (E–H).

From Downy Mildew-Infected Plants				From Control Healthy Plants			
**A**	**Ovaries**			**E**	**Ovaries**		
**Host**	**Examined**	**Infectious**	**%**	**Host**	**Examined**	**Infectious**	**%**
Cucumber	82	15	18.3	Cucumber	40	0	0.0
Melon	10	3	30.0	Melon	10	0	0.0
Squash	37	14	37.8	Squash	30	0	0.0
**Total**	**129**	**32**	**24.8**	**Total**	**80**	**0**	**0.0**
**B**	**Fruit seed cavity**			**F**	**Fruit seed cavity**		
**Host**	**Examined**	**Infectious**	**%**	**Host**	**Examined**	**Infectious**	**%**
Cucumber	316	22	7.0	Cucumber	100	0	0
Melon	116	8	6.9	Butternut gourd	30	0	0
Pumpkin	29	3	10.3	Pumpkin	100	0	0
Squash	49	1	2.0	Squash	20	0	0
**Total**	**510**	**34**	**6.7**	**Total**	**160**	**0**	**0.0**
**C**	**Seeds**			**G**	**Seeds**		
**Host**	**Sown**	**Infected plants**	**%**	**Host**	**Sown**	**Infected plants**	**%**
Cucumber	400	1	0.25	Cucumber	200	0	0
Butternut gourd	400	4	1	Butternut gourd	200	0	0
Squash	400	6	1.5	Squash	200	0	0
**Total**	**1200**	**11**	**0.92**	**Total**	**600**	**0**	**0.0**
**D**	**Hypocotyls**			**H**	**Hypocotyls**		
**Host**	**Examined**	**Infectious**	**%**	**Host**	**Examined**	**Infectious**	**%**
Cucumber	150	1	0.7	Cucumber	50	0	0
Melon	150	2	1.3	Butternut gourd	50	0	0
Squash	150	4	2.7	Squash	50	0	0
**Total**	**450**	**7**	**1.6**	**Total**	**150**	**0**	**0.0**

The downy mildew-infected plants were grown in net-houses #2, 3, 5 and 6 while the healthy plants were grown in greenhouse #3.

**A, E** - Infectivity of crushed ovaries to detached leaves of cucumber and/or butternut gourd.

**B, F** - Infectivity of crushed fruit seed-cavity tissue to detached leaves of cucumber and/or butternut gourd.

**C, G** - Vertical transmission of *P. cubensis* from seeds to the next plant generation.

**D, H** - Infectivity of crushed hypocotyls to detached leaves of cucumber and/or butternut gourd.

**Figure 2:**
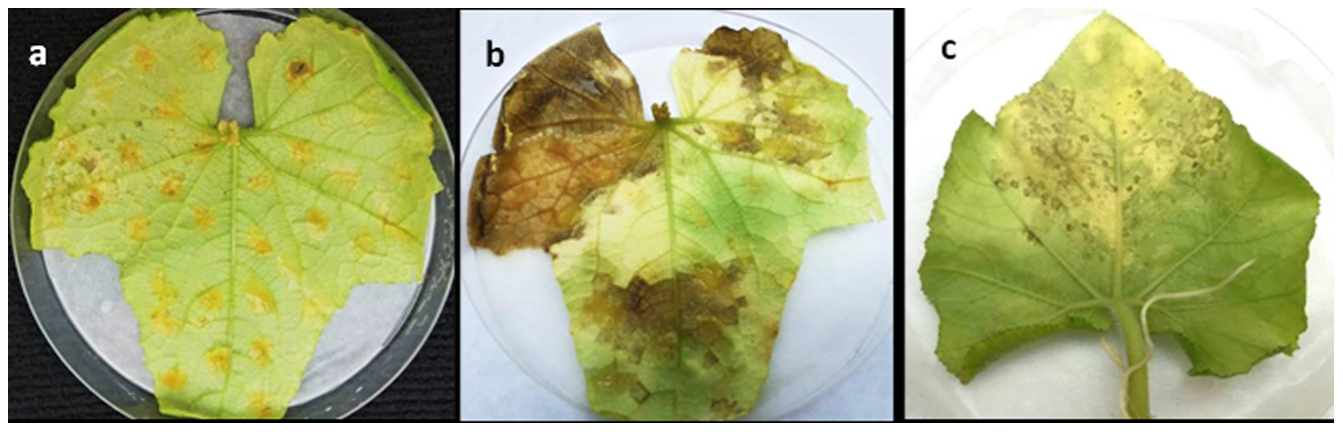
Tissue homogenate taken from the seed-cavity of butternut gourd or cucumber fruits are infective to detached leaves of cucumber and butternut gourd. **a** downy mildew starting to develop in a detached leaf of cucumber at 1 week after inoculation; **b**, **c**, downy mildew developed in a detached leaf of cucumber and butternut gourd, respectively at 3 weeks after inoculation.

A total of 510 fruits of cucumber, melon, pumpkin or squash were collected during 2012–2014. Fruits were surface sterilized, cut open in two halves, seeds (when available) removed, and the seed cavity tissue crushed and inoculated onto detached leaves. Thirty four fruits (6.7%) were infectious, producing typical downy mildew on the detached leaves of cucumber and/or butternut gourd (Table 1B; Fig. 2). The isolates recovered from seed cavity of cucumber and melon belonged to pathotype 3 mating type A1, while those recovered from pumpkin and squash belonged to pathotype 6 mating type A2.

**Figure 3:**
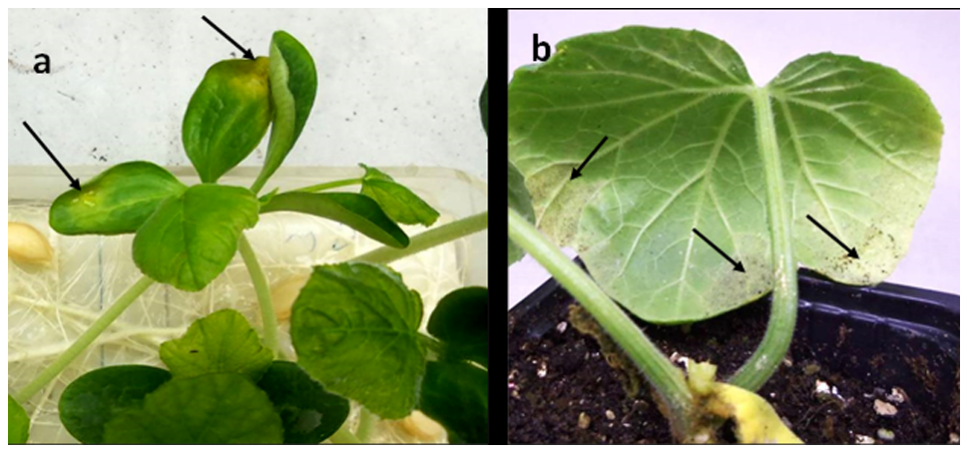
Seed transmission of downy mildew caused by *Pseudoperonospora cubensis* in butternut gourd (*Cucurbita moschata*). **a** symptoms (arrows) on cotyledons at 10 days after germination. **b** symptoms with sporulation (arrows) on the first true leaf at 2 weeks after transplanting to soil.

Mature seeds were taken from fruits of cucumber, butternut gourd or squash, surface-sterilized, planted (20 seeds per fruit, 20 fruits from each species) in sterile soil mixture in pots and grown for one month at 25°C with 12 h light/day. As shown in Table 1C, one cucumber, four butternut gourd and six squash plants developed downy mildew symptoms with sporulation of *P. cubensis* (Fig. 3).

In a similar experiment, seeds were planted in sterile soil and grown in greenhouse #3. At 2 weeks after germination no disease was seen on the cotyledons. The hypocotyls were taken, surface-sterilized, homogenized in sterile water and inoculated onto detached leaves. Seven out of the 450 hypocotyls tested (1.6%) were infectious, producing downy mildew on detached leaves of cucumber and/or butternut gourd (Table 1D).

In parallel experiments, ovaries, fruits and seeds were collected during 2012–2014 from healthy plants growing in glasshouse #3 in which the controlled dry atmosphere prevented downy mildew development. None of the 80 ovaries, 160 fruits or 150 hypocotyls was infectious to detached leaves of cucumber or butternut gourd (Table 1 E, F, H). No seed of the 600 seeds sown developed downy mildew symptoms (Table 1 G).

### Microscopy

**Figure 4:**
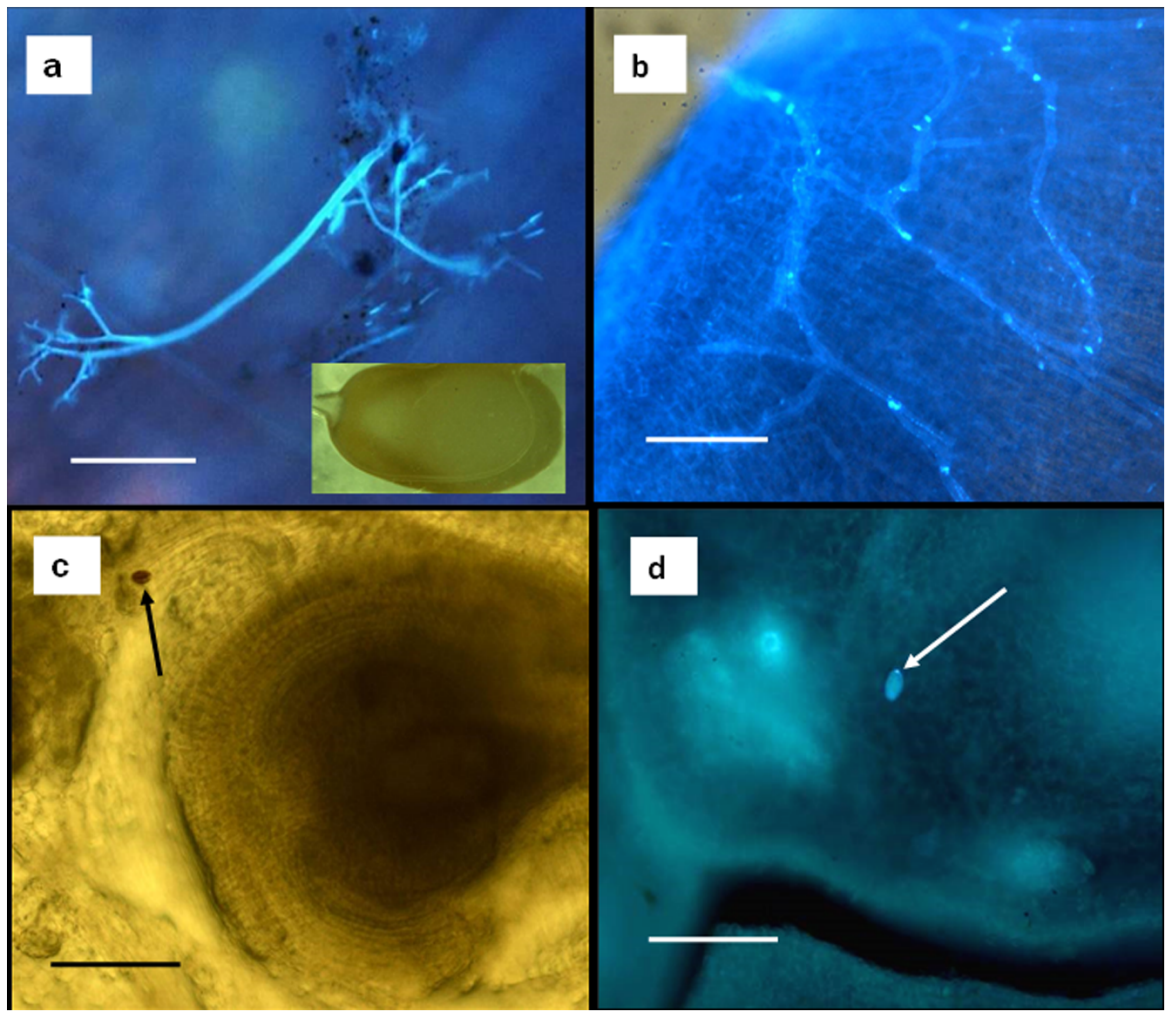
*P.cubensis* in embryos and ovaries of cucumber and butternut gourd. **a** sporangiophores with sporangia on a cucumber embryo (insert: a cucumber seed, actual size  = 9 mm). **b** mycelium in an embryo of butternut gourd. **c** and **d** sporangia (arrow) in the ovary of cucumber near an ovule. Bar in **a** and **c** = 100 µm; in **b** and **d** = 200 µm.

A rare observation of *P.cubensis* sporulating on embryo of cucumber is shown in Fig. 4a. Mycelium inside the embryo of butternut gourd is shown in Fig. 4b. Sporangia inside ovaries of cucumber, adjacent to the ovules, are shown in Fig. 4c and 4d.

### PCR assays for *P. cubensis* in ovaries and seed cavity

About 49% of the ovaries (39 out of 80) (Table 2A) and 55% of the flower peduncles (6 out 11) (data not shown) reacted positively when tested with primers Set 1. None of the 45 healthy ovaries has tested positive (Table 2B). Of the 301 fruits examined, 35 (11.6%) tested positive with primers Set 1 (Table 2C). All 120 healthy fruits from greenhouse #3 tested negatively to *P. cubensis* (Table 2D).

**Table 2:** PCR assays showing the occurrence of *Pseudoperonospora cubensis* amplicons in ovaries and fruits of cucurbits.

**A. Ovaries from infected plants**		
**Host**	**Ovaries**	**Primer Set 1**
Cucumber	38	22
Squash	37	16
Melon	5	1
**Total**	**80**	**39**
**B. Ovaries from healthy plants**		
**Host**	**Ovaries**	**Primer Set 1**
Cucumber	15	0
Squash	15	0
Melon	15	0
**Total**	**45**	**0**
**C. Seed cavity of fruits from infected plants**		
**Host**	**Fruits**	**Primer Set 1**
Cucumber	236	26
Butternut gourd	28	4
Pumpkin	37	5
**Total**	**301**	**35**
**D. Seed cavity of fruits from healthy plants**		
**Host**	**Fruits**	**Primer Set 1**
Cucumber	80	0
Butternut gourd	20	0
Pumpkin	20	0
**Total**	**120**	**0**

Interestingly, in 12 cucumber fruits whose proximal and distal ends were tested separately, 5 tested positive, all at the proximal end (stem end) of the fruit. Similarly, in 6 squash fruits all tested positively, all at the proximal end of the fruit. PCR assays conducted with pistil tissue of squash female flowers resulted with no signal, suggesting that the pathogen moves into the fruit from the stem side and not from the pistil side. Indeed, PCR assays revealed the occurrence of *P. cubensis* in stems of cucumber, melon, squash and pumpkin. Often, petioles and stem homogenates were infectious to detached leaves of cucumber and/or butternut gourd.

### PCR assays for *P.cubensis* in seed integuments and embryos

**Figure 5:**
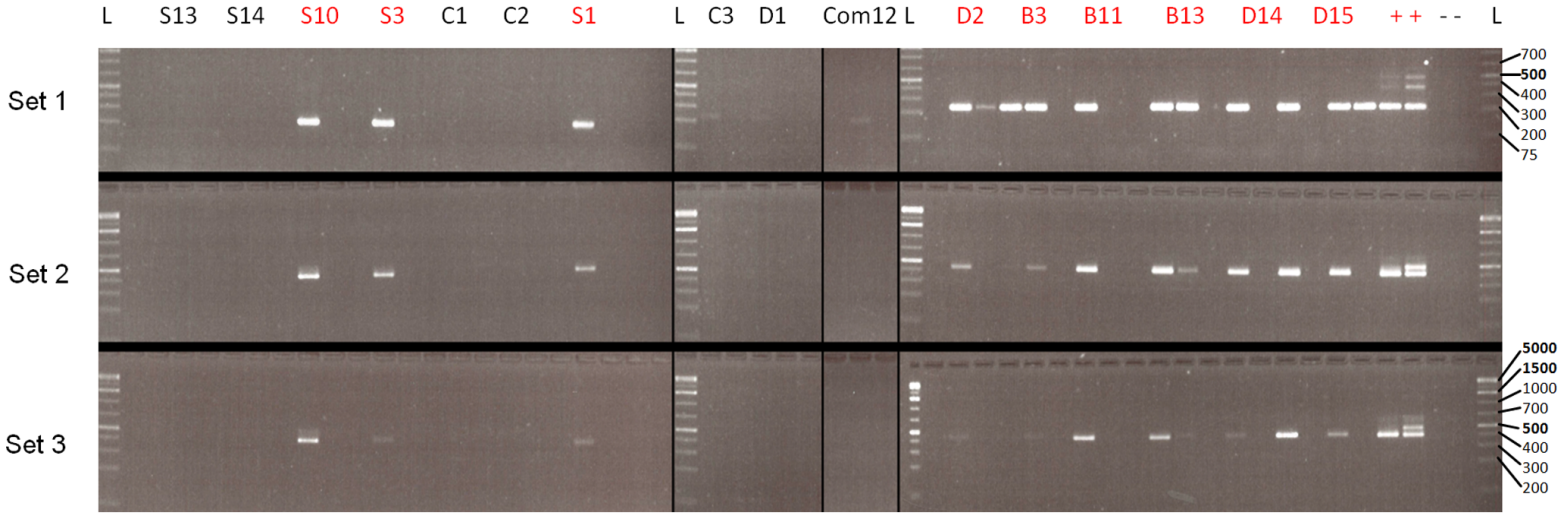
PCR analysis of 16 seed samples, 3 seeds per sample, one slot per seed. In the left part- embryos were tested. In the two middle parts, weak bands with only primer set 1, are seen. In the right part integuments were tested (seeds were empty). L = 1 kb plus ladder (Fermentas). S =  squash Arlika. C =  cucumber. Com12 =  cucumber cv. Marco, Nickerson Zwaan. D =  butternut gourd. B =  squash Beruti. +  =  positive control (left: NY425 – clade 2, right: NY427 – clade 1 (Runge et al. 2011)). -  =  negative control (sterile water).

PCR assays showed that *P. cubensis* occurs in seeds collected from downy mildew-infected plants (Fig. 5, Tables 3–5). In Fig. 5, three seeds per entry were used, one slot per seed. A positive reaction to primer Set 1(detecting all 3 clades) was detected in 10 out of 15 seed samples. In 6 samples, only one seed responded positively whereas in 4 samples, 2 out of 3 seeds responded positively, indicating on the heterogeneity of infection in the seeds in a single fruit. The same number of samples responded to primer Set 2 (detecting clades 1 and 2), but with only one seed out of three responding positively. Eight samples responded to primer set 3 (detecting clade 2), one seed per sample. Sequencing of 6 randomly chosen PCR products confirmed the identity of the organism as being *P. cubensis*.

**Table 3:** PCR assays showing the occurrence of *Pseudoperonospora cubensis* amplicons in individual seeds of cucurbits.

Fruit	Seed	ITS - Test	ITS - Test	cox2	cox2	All 3 clades		Clades 1 and 2		Clade 1	
Name	Number	Integument	Embryo	Integument	Embryo	Integument	Embryo	Integument	Embryo	Integument	Embryo
**B2**	1	**1**	**1**	0	0	**1**	0	0	0	0	0
**B2**	2–10	**1**	**1**	0	0	0	0	0	0	0	0
**D2**	1–6	**1**	**1**	0	0	0	0	0	0	0	0
**D2**	7	**1**	**1**	0	0	0	**1**	0	**1**	0	**1**
**D2**	8–10	**1**	**1**	0	0	0	0	0	0	0	0
**D3**	1–10	**1**	**1**	0	0	0	0	0	0	0	0
**D4**	1–10	**1**	**1**	0	0	0	0	0	0	0	0
**D5**	1–3	**1**	**1**	0	0	0	0	0	0	0	0
**D5**	4	**1**	**1**	0	0	0	**1**	0	1	0	**1**
**D5**	5–8	**1**	**1**	0	0	0	0	0	0	0	0
**D5**	9	**1**	**1**	0	0	**1**	0	**1**	0	**1**	0
**D5**	10	**1**	**1**	0	0	0	0	0	0	0	0
**D6**	1	**1**	**1**	0	0	**1**	0	**1**	0	1	0
**D6**	2	**1**	*0	0	0	0	0	0	0	0	0
**D6**	3	**1**	*0	0	0	**1**	0	**1**	0	**1**	0
**D6**	4–10	**1**	*0	0	0	0	0	0	0	0	0
**D7**	1–10	**1**	**1**	0	0	0	0	0	0	0	0
**H2O**		0	0	0	0	0	0	0	0	0	0
**"1953"**		**no positive controls used**		**1**	**1**	**1**	**1**	**1**	**1**	**1**	**1**
							**additional positive control "21226"**			**1**	**1**
			*empty		**Total**	**5**	**3**	**4**	**3**	**5**	**4**

The seeds were taken from one fruit of squash (B) and 6 fruits of butternut gourd (D) (10 seeds per fruit). 1 =  test positive; 0 = test negative.

**Table 4:** PCR assays showing the occurrence of *Pseudoperonospora cubensis* in individual seeds of cucumber.

A. Seeds from downy mildew-infected plants			
Fruit Name	Seed Number	Clade 1	
		Integument	embryo
**C1**	**1**	**0**	**0**
	2	**1**	0
	3–5	0	0
**C2**	1–4	0	0
**C3**	1–5	0	0
**C4**	1	0	**1**
	2–4	0	0
	5	**1**	**1**
**C5**	1, 2, 4	0	0
	3	0	**1**
	5	**1**	0
**C6**	1, 2, 3, 5	0	0
	4	0	**1**
**C7**	1–5	0	0
**C8**	1–5	0	0
**C9**	1–5	0	0
**C10**	1–5	0	0
**C11**	1, 2, 3, 5	0	0
	4	0	**1**
**C12**	1, 3, 4, 5	0	0
	2	**1**	0
**C13**	1	0	**1**
	2, 3, 4	0	0
	5	**1**	0
**C14**	1–3	0	0
**C15**	1–3	0	0
**Total**	**70**	**5**	**6**

B. Seeds from healthy plants			
Fruit Name	Seed Number	Clade 1	
		Integument	embryo
**C1**	1–5	0	0
**C2**	1–5	0	0
**C3**	1–5	0	0
**C4**	1–5	0	0
**C5**	1–5	0	0
**C6**	1–5	0	0
**C7**	1–5	0	0
**C8**	1–5	0	0
**C9**	1–5	0	0
**C10**	1–5	0	0
**Total**	**50**	**0**	**0**

A. Fruits taken from downy mildew-infected plants. B. Fruits taken from healthy plants.

B. 1 =  test positive; 0 = test negative.

**Table 5:** PCR assays showing the occurrence of *Pseudoperonospora cubensis* in individual seeds of bottle gourd (*Lagenaria vulgaris*).

		A. Fruits from downy mildew-infected plants					
		All 3 clades		Clade 1 and 2		Clade 1	
Fruit name	Seed Number	Integument	Embryo	Integument	Embryo	Integument	Embryo
**Lag1**	5	0	0	**1**	0	**1**	0
	1–4, 6–10	0	0	0	0	0	0
**Lag2**	2	0	0	0	0	**1**	0
	7	0	0	0	0	**1**	0
	9	0	0	0	0	**1**	**1**
	1.3–5, 8, 10	0	0	0	0	0	0
**Lag3**	4	0	0	0	0	0	**1**
	5	0	0	0	0	**1**	**1**
	7	0	0	**1**	0	0	**1**
	9	0	0	**1**	0	**1**	**1**
	1–3, 6, 8, 10	0	0	0	0	0	0
**Lag4**	2	0	0	0	0	**1**	0
	8	0	0	**1**	0	0	0
	1, 3–7, 9, 10	0	0	0	0	0	0
**Lag5**	1–10	0	0	0	0	0	0
**Lag6**	1–10	0	0	0	0	0	0
**Lag7**	3	0	0	**1**	0	**1**	0
	4	0	0	0	0	**1**	**1**
	5	0	0	**1**	0	**1**	0
	1, 2, 6–10	0	0	0	0	0	0
**Lag8**	1–10	0	0	0	0	0	0
**Lag9**	1–10	0	0	0	0	0	0
**Lag10**	5	0	0	0	0	**1**	**1**
	6	0	0	0	0	**1**	**1**
	7	0	0	0	0	**1**	**1**
	9	0	0	0	0	0	**1**
	10	0	0	0	0	**1**	**1**
	1–4, 8	0	0	0	0	0	0
**Total**	**100**	**0**	**0**	**6**	**0**	**14**	**11**

		B. Fruits from healthy plants					
		All 3 clades		Clade 1 and 2		Clade 1	
Fruit name	Seed Number	Integument	Embryo	Integument	Embryo	Integument	Embryo
**Lag1**	1–10	0	0	0	0	0	0
**Lag2**	1–10	0	0	0	0	0	0
**Lag3**	1–10	0	0	0	0	0	0
**Lag4**	1–10	0	0	0	0	0	0
**Lag5**	1–10	0	0	0	0	0	0
**Lag6**	1–10	0	0	0	0	0	0
**Lag7**	1–10	0	0	0	0	0	0
**Lag8**	1–10	0	0	0	0	0	0
**Lag9**	1–10	0	0	0	0	0	0
**Lag10**	1–10	0	0	0	0	0	0
**Total**	**100**	**0**	**0**	**0**	**0**	**0**	**0**

A- Fruits taken from downy mildew-infected plants. B- Fruits taken from health plants.

1 =  test positive; 0 = test negative.

Table 3 presents the molecular detection analyses obtained for 10 seeds of 7 fruits: B2 of squash and D2–D7 of butternut gourd. The occurrence of *P. cubensis* was tested separately in the integuments and the embryo (see Methods and Material). Two embryos (D2/7, D5/4) and three integuments (D5/8, D6/1 and D6/3) were tested positive for *P. cubensis*. One seed B2/1 tested positive for primers Set 1 only.

In another assay (Table 4), the integuments and embryo of 70 individual seeds (from 15 fruits) of cucumber were analyzed with primer Set 3 that amplifies clade 1 of *P. cubensis*. The integuments of 5 seeds and the embryo of 6 seeds tested positive. In only one seed (C4/5) both the integuments and the embryo tested positive (Table 4A), suggesting that the pathogen may colonize the integuments, the embryo, or both. No amplicons of *P. cubensis* were detected in seeds taken from healthy plants (Table 4B).

Similar results were obtained with seeds of bottle gourd (*Lagenaria vulgaris*) (Table 5). The integuments and embryo of 100 individual seeds (10 seeds/fruit) were analyzed with all three primer sets of *P. cubensis*. Eight seeds tested positive with primer set 3 in both the integument and embryo, 6 seeds tested positive with primer set 2 in the integument but not the embryo, and no seed was positive with primer set 1 (Table 5A). No amplicons of *P.cubensis* were detected in seeds taken from healthy plants (Table 5B).

In pumpkin, we tested 42 embryos from 5 infected fruits with primer sets 2 and 3. Two out of 5 fruits had PCR-positive embryos. In one fruit, 4 out of 10, and in the second 1 out of 10, tested positive with both primers sets 2 and 3. Thirty embryos from 4 healthy fruits were all PCR-negative.

## Discussion

*P. cubensis* is a foliar pathogen of *Cucurbitaceae* with world-wide distribution. In the past decade major changes occurred in the population structure of this oomycete. In Israel, two major changes took place: in 2002 a new pathotype, number 6, appeared [8] and in 2010 a new mating type, A2, showed up [9]. In the USA a new race appeared in 2004 capable of destroying long-lasting resistant cucumber cultivars [16]. In Italy, a new pathotype, number 5, appeared in 2003 [17], and in the Czech Republic many new pathotype combinations appeared recently [18].

Runge et al [13] performed a molecular comparison between pre-2004 and post-2004 isolates of *P. cubensis*. They suggested that the new post-2004 genotypes have migrated (by man carrying leaf material) from South East Asia (Korea, Japan) to Europe and thereafter to the USA.

Another vehicle for such a migration could be a man carrying fruits or seeds of cucurbits, or commercial trade of fruits and seeds. We show here that fruits of cucurbits, collected from downy mildew-infected plants are symptomless but may carry mycelium and sporangia of *P. cubensis*. Fruit slices, or seed-cavity tissue homogenates made from such fruits, produced typical downy mildew symptoms with sporulation of *P. cubensis* when applied to detached healthy leaves of cucurbits. *P. cubensis* similarly occurs in symptomless stems, petioles and ovaries of infected plants. PCR assays showed that the pathogen occurs in peduncles of female flowers and at the stem end, not in the petal end, of the ovary or fruit, nor in the pistil of the flower. This suggests that penetration of the pathogen into the ovary, and thereafter into the fruit, occurs from the leaf into the stem and then through the peduncle of the female flower into the ovary.

*P. cubensis* was found to also occur in seeds of cucurbits. We confirmed it by microscopy, infectivity of crushed seeds to detached leaves, and species-specific PCR assays. All implicated that *P. cubensis* may be transmitted by seed.

Indeed, seeds collected from infected plants produced infected plants with typical symptoms and sporulation of *P. cubensis*. Vertical seed transfer of downy mildew occurred in cucumber (0.25%), butternut gourd (1%) and squash (1.5%). Hypocotyls produced by such seeds were infectious (1.6%) when crushed and inoculated onto detached leaves of cucumber.

These findings may now offer a new explanation to the global structural changes in the pathogen population. Cucurbit fruits (probably ornamental squash or pumpkin) were imported for commercial purposes, or transported by man, from South East Asia into Europe and/or Israel. The fruits were collected from infected plants and probably carried *P. cubensis* belonging to pathotype 6 mating type A2. Our survey [19] approved the occurrence in China of isolates that belong to the pathotype 6 mating type A2. Another survey [20] showed a clear preference of isolates belonging to pathotype 6 mating type A2 to *Cucurbita* species (pumpkin, squash, butternut gourd). It could be that the original migration took place from China and not necessarily from Korea or Japan, or that the population of *P. cubensis* in China, Korea and Japan are composed of similar genotypes. The new immigrants may have undergone sexual mating with the prevailing local isolates that belong to pathotype 3 mating type A1. Oospores were produced and the new recombinant offspring isolates were probably capable of attacking new hosts (see [9]). The new isolates may have similarly migrated to the USA [21] where they were capable of breaking down the long-lasting resistance of cultivars of cucumber.

Seed infection by downy mildew agents is common, but seed transmission of downy mildew diseases is rare and happens in a rather low proportion. Seeds collected from sunflower plants systemically infected with the homothallic oomycete *Plasmopara halstedii* contain mycelia, sporangia, oogonia and oospores of the pathogen in the inner surface of the pericarp and embryo. Only one out 276 plants grown from such seeds (0.36%) produced typical systemic infection [22]. Seed of *Camelina sativa* were reported [23] to carry sporangia of *Hyaloperonospora camelinae* on their surface and produced (no surface disinfection) 96% infected plants. Garibaldi et al [24] and Djalali et al [25] showed that basil seeds are infected with *Peronospora belbahrii*. The late blight oomycete pathogen *Phytophthora infestans* may infect seeds of tomato. Such infection occurs when fruits are inoculated on their stem scar with mixed A1+A2 sporangia [26]. The infected seeds harbor mycelia, sporangia, and oospores of the pathogen in the pericarp and embryo [26] with only one out of about 1000 seedlings showing late blight infection (Rubin and Cohen, unpublished data). Landa et al [27] demonstrated that *Peronospora arborescens*, the causal agent of downy mildew in opium poppy, can be transmitted in seeds and that commercial seed stocks collected from crops with high incidence of the disease were infected but produced a rather low incidence of infected plants. Quinoa seeds were reported to harbor oospores of the downy mildew agent *Peronospora variabilis* [28].

In conclusion, this paper demonstrates that under experimental conditions, *P. cubensis* can be transmitted from infected plants to their pedigree seeds. This fact calls for immediate action to elucidate if seed transmission may also occur in agricultural practices. Further studies are required to better understand the mechanisms by which fruits and seeds become contaminated. More research should be devoted to further confirm the vertical transmission of the pathogen to the next generation of cucurbits. The close relationships between *P. cubensis* and *P. humuli* amplicons need to be studied further. Special care should be taken by growers not to use seeds from downy mildew-infected plants.

## Supporting Information

Table S1 **Three sets of primers used to identify** ***Pseudoperonospora cubensis*** **in sporangia and plant material of cucurbits.**
(DOCX)

Table S2 **Temperatures and cycling times of PCR reactions used to identify** ***Pseudoperonospora cubensis*** **in sporangia and plant material of cucurbits.** First PCR program for primer pair *cox*2-F and *cox*2-R. Second PCR program for species-specific primers given in Table 1. Steps 2-4 were repeated 35 times.
(DOCX)
