# Supplementary material for: Seed Transmission of Pseudoperonospora cubensis
Source: PLoS One. 2014 Oct 17;9(10):e109766. doi: 10.1371/journal.pone.0109766 (PMC4201460; doi:10.1371/journal.pone.0109766)
Supplement: Table S1 — Three sets of primers used to identify Pseudoperonospora cubensis in sporangia and plant material of cucurbits. (DOCX) [file pone.0109766.s001.docx]

**Table S1**

Three sets of primers used to identify *Pseudoperonospora cubensis* in sporangia and plant material of cucurbits.

| **Primer Set** | **Forward** | **Reverse** | **Specificity** |
| --- | --- | --- | --- |
| **1** | GGATGAAGTAATTGATCCTATC | GTAATTAATACTCGAATATGG | *P. cubensis* clade 1 and clade 2, and *P. humuli* |
| **2** | TAATTGTAGTTACAGTATTC | GTAAAACATCAGAAGCTGTG | *P. cubensis* clades 1 and clade 2 |
| **3** | TAATTGTAGTTACAGTATTC | GTAATTAATACTCGAATATGG | *P. cubensis* clade 1 |
