# Supplementary material for: Seed Transmission of Pseudoperonospora cubensis
Source: PLoS One. 2014 Oct 17;9(10):e109766. doi: 10.1371/journal.pone.0109766 (PMC4201460; doi:10.1371/journal.pone.0109766)
Supplement: Table S2 — Temperatures and cycling times of PCR reactions used to identify Pseudoperonospora cubensis in sporangia and plant material of cucurbits. First PCR program for primer pair cox2-F and cox2-R. Second PCR program for species-specific primers given in Table 1. Steps 2-4 were repeated 35 times. (DOCX) [file pone.0109766.s002.docx]

**Table S2**

Temperatures and cycling times of PCR reactions used to identify *Pseudoperonospora cubensis* in sporangia and plant material of cucurbits.

First PCR program for primer pair *cox*2-F and *cox*2-R. Second PCR program for species-specific primers given in Table 1. Steps 2-4 were repeated 35 times.

|  | **First PCR** | | **Second PCR** | |
| --- | --- | --- | --- | --- |
|  | **Temperature (°C)** | **Time (min)** | **Temperature(°C)** | **Time (min)** |
| 1. Initial Denaturation | 95 | 4:00 | 95 | 4:00 |
| 1. Denaturation | 95 | 0:40 | 95 | 0:40 |
| 1. Annealing | 48.5 | 0:40 | 52.5 | 0:40 |
| 1. Elongation | 72 | 0:40 | 72 | 1:00 |
| 1. Final Elongation | 72 | 4:00 | 72 | 4:00 |
